# Effects of C-S-H Seed Prepared by Wet Grinding on the Properties of Cement Containing Large Amounts of Silica Fume

**DOI:** 10.3390/polym16192769

**Published:** 2024-09-30

**Authors:** Shiheng Wang, Peng Zhao, Yaogang Tian, Jianan Liu

**Affiliations:** School of Materials Science and Engineering, Chang’an University, Xi’an 710061, China; 2019031010@chd.edu.cn (S.W.); ygtian@chd.edu.cn (Y.T.)

**Keywords:** high-performance concrete, C-S-H seed suspension, stability, mechanical properties

## Abstract

This study aimed to utilize the hydration characteristics of cement through wet grinding techniques to efficiently and conveniently prepare a stable C-S-H seed suspension, providing key parameters and a scientific basis for their large-scale production, which ensures the stability of the C-S-H suspension during production, transportation, and application. This preparation aimed to mitigate the adverse effects of high-volume silica fume on the early mechanical properties of high-performance cement concrete. The properties of C-S-H seed were characterized in detail by SEM, XRD, and TD. In the concrete performance test, silica fume was used to replace part of the cement, and different contents of C-S-H seed were added to test its effect on the compressive strength of concrete, with XRD and SEM used to analyze the performance differences. The results show that the particle size and hydration degree of cement no longer developed after 90 min of wet grinding. Polycarboxylate ether (PCE) superplasticizer can increase the fluidity of the crystal C-S-H seed suspension when the content exceeds 1.5%. When the content of PCE exceeded 2%, the C-S-H seed suspension precipitated. Adding 5% C-S-H seed can increase the compressive strength of cement concrete by 10% under the condition of reducing the amount of cement and increasing the amount of silica fume. And Ca(OH)_2_ (CH) was produced by cement hydration consumed by silica fumes to generate C-S-H gel, by which the concrete became denser with more strength. However, when the amount of C-S-H seed exceeded 7%, the compressive strength of the concrete decreased.

## 1. Introduction

To expedite the strength development of high-performance cement concrete, high-temperature steam curing is commonly employed. This method can achieve the desired concrete strength within 6 to 10 h of steam curing. Although steam curing significantly reduces curing time and enhances production efficiency, it often results in larger voids between the cement hydration products. Then, these pores remain unfilled, leading to strength regression and negatively impacting durability. To address this issue, silica fume is added to high-strength concrete to replace part of the cement. This addition not only increases the dense quality and reduces the shrinkage of the concrete but also enhances the utilization of mineral waste like silica fume and lowers production costs. However, when the content of silica fume is high, it can adversely affect concrete strength, limiting its widespread application in concrete. Due to the drawbacks of steam curing, researchers have proposed adding nanomaterials as nucleation points for cement hydration in early strength studies of concrete. This approach induces the formation and accumulation of calcium silicate hydrate (C-S-H) gel, further accelerating the dissolution of silicate phases and the generation of hydration products, thereby shortening the induction period of cement hydration and enhancing the early strength of cement concrete. Nano-crystalline materials can be classified into two types based on their mechanism of action. The first type includes nanoparticles such as nano-TiO_2_ [1,2], nano-Fe_2_O_3_ [3], nano-ZrO_2_ [4], and carbon nanotubes [5]. These nanoparticles provide nucleation sites for C-S-H gel and other hydration products during cement hydration, thereby accelerating the process. The second type consists of reactive nanoparticles such as nano-SiO_2_ [6], nano-Ca(OH)_2_ [7], and nano-CaCO_3_ [8]. These not only provide nucleation sites for hydration products but also participate in the cement hydration process. Among the various nano-crystalline materials, synthetic nano C-S-H seeds are particularly noteworthy. Due to their similar chemical composition to the primary hydration product C-S-H gel, they do not alter the chemical composition of the pore solution when introduced into the cement paste, making them an excellent nucleation substrate for C-S-H gel [9].

C-S-H is an amorphous substance formed by the hydration of C_3_S and C_2_S in cement, accounting for more than 50% of hydration products [10,11]. It is one of the primary sources of strength in cementitious materials. In the 1990s, researchers introduced synthetic C-S-H to address the issue of limited diffusion of hydration products during cement hardening [12,13]. Studies have shown that C-S-H can overcome the localized densification of hydration products in cement, leading to a uniform distribution and dense structure. This reduces the capillary pore content and refines the pore distribution in the cement paste. This effect is attributed to the preferential adsorption interface effect of C-S-H seed, which promotes the preferential crystallization of ions on its surface, thereby accelerating C_3_S hydration, shortening the induction period, and increasing the degree of hydration. At the cement paste–aggregate interface, C-S-H seed significantly inhibits the volume of CH crystals and promotes the formation of C-S-H gel, thereby reducing the porosity of the interfacial zone. Thomas [14] studied the effect of different amounts of C-S-H seed on the hydration of silicate cement and pure C_3_S. The study showed that the addition of C-S-H seed shortened the hydration induction period, advanced the main peak of C_3_S hydration exothermicity, and increased the early hydration heat release. This accelerates the formation of hydration products, thus significantly enhancing the early performance of cementitious materials. C-S-H seed accelerate C_3_S hydration by continuously combining with Ca^2+^ in solution and can directly serve as secondary nucleation or growth points for hydration products. When hydration products grow on C-S-H seed rather than on clinker particles, it reduces the inhibitory effect on C_3_S dissolution, thus accelerating cement hydration. Therefore, C-S-H seed allows hydration products to grow in the pore solution without covering the surface of cement particles, resulting in less hindrance to dissolution. This mechanism of promoting hydration by serving as nucleation sites for C-S-H precipitation is known as “seed technology”, and these nanoparticles are referred to as “C-S-H nucleating agents” or “C-S-H seed”. The early strength enhancement brought by C-S-H seed does not negatively impact later strength and can effectively improve the early mechanical properties of concrete, compensating for the early strength reduction caused by fly ash.

The effectiveness of nucleating agents in enhancing the early strength of cementitious materials has gained widespread recognition. However, the high cost and complexity of chemically synthesized C-S-H seed are significant barriers [15,16,17]. To devise an efficient and straightforward method for preparing C-S-H seed, Li [18] utilized wet grinding of cement, inducing slight hydration during the process, which resulted in high reactivity, nucleation induction effects, and filling effects. Tan [19] prepared C-S-H seed by fully hydrating C3S and cement through wet grinding. The primary hydration product, C-S-H gel, was physically ground in a liquid phase environment containing ion-promoting agents and surface modifiers, reducing the C-S-H seed to nanoscale size. In an effort to enhance the comprehensive utilization of solid waste and reduce raw material costs, researchers have employed reactive solid wastes such as blast furnace slag [20], carbide slag, kaolin [21,22,23], and fly ash [24,25,26]. These wastes are finely ground into slurry through a wet grinding process. The increased particle size, disruption of the original molecular structure, and exposure of active reaction sites accelerate the dissolution of Si, Al, and Fe ionic groups on the fractured particle surfaces. Solid wastes can hydrate to produce C-S-H seed exhibiting high pozzolanic activity. Their use significantly improves the early compressive strength, provides a filling effect, and enhances the pore structure of these materials. The influence becomes more pronounced with extended grinding time. Their early strength mechanism is based on the nucleation effect, which directs the migration of various mineral ions during cement hydration and provides nucleation centers for the growth of various hydration products or crystals. This accelerates the growth rate of hydration products and enhances the degree of cement hydration [27]. Common methods for preparing C-S-H seed, such as hydrothermal synthesis, involve complex processes, strict conditions, and difficult-to-control parameters. Additionally, the stability of the synthesized C-S-H gel is often poor, posing challenges for industrial production and application [28].

This study aims to promote the practical application of C-S-H seeds, providing key parameters and a scientific basis for their large-scale production. By preparing C-S-H seed suspension through a wet grinding process, we simplify the manufacturing process, save energy, and address the issues of easy aggregation and poor dispersion characteristics of C-S-H seeds. This ensures the stability of the C-S-H suspension during production, transportation, and application. This paper used a wet grinding process to prepare a C-S-H seed suspension with good fluidity and stability under the action of a PCE. First, the effect of wet grinding time on the particle size and hydration degree of C-S-H seed was first studied. Secondly, the effect of the polycarboxylate water reducer on the kinematic viscosity and zeta potential of C-S-H seed suspension was studied. Finally, in the study of concrete mechanical properties, the effect of C-S-H seed amount on the strength of concrete was studied, and its microscopic characteristics were analyzed by SEM, XRD, and TD.

## 2. Materials and Methods

### 2.1. Materials

The Portland cement (P.O. 42.5), S95 ground granulated blast slag (GGBS), and SF90 silica fume (SF) used in this paper were tested by X-ray fluorescence (XRF), and results are shown in Table 1. The PCE used was Sika^®^ Visco Crete^®^ 325C.

### 2.2. Sample Preparation

#### 2.2.1. Preparation of C-S-H Seed

**Figure 1:**
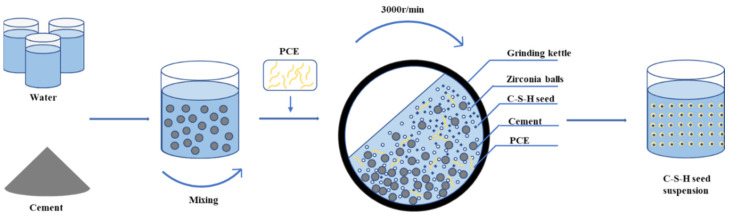
C-S-H seed preparation process.

C-S-H seed was prepared by the wet grinding process, with a water–cement ratio of 3:1, as a high concentration can reduce the distance between each C-S-H seed and improve the repulsion, which is conducive to the stability of C-S-H gel suspension [29]. Zirconia balls with a diameter of 1.0 mm were selected as grinding media by ball mill. The mass ratio of zirconia balls to raw materials was 5:1, and increasing the pressure inside the grinding kettle helped to grind and disperse C-S-H seed [30,31]. First, the raw materials were mixed and stirred with water for 1 d to fully react with hydration, and then the PCE was added and wet-milled for 120 min to obtain C-S-H seed. The preparation process is shown in Figure 1.

#### 2.2.2. Concrete Sample Preparation

The content levels of C-S-H seed were 0%, 1%, 3%, 5%, 7%, 9%, and 11% of the mass of cementitious materials in concrete; the concrete mix proportions are shown in Table 2. Concrete specimens of 100 mm × 100 mm × 100 mm were prepared and cured according to standard GB/T 50081-2019 [32]. Steam curing was carried out according to standard GB/T 13476-2023 [33], and the compressive strength was tested.

According to standard GB/T 17671-2021 [34], 40 mm × 40 mm × 40 mm cement paste was prepared, and the C-S-H seed content levels were 0%,1%, 3%, 5%, 7%, 9%, and 11% of the total mass of the cementitious material. The mix ratio is shown in Table 3. After steam curing according to standard GB/T 13476-2023 [33], the specimens were crushed into small pieces, immersed in anhydrous ethanol for about 3 h, and dried in an environment of 100 °C for 24 h after the hydration reaction stopped. A part of the fragments was selected for SEM testing, and the rest was ground and used for other tests.

### 2.3. Experiment Method

#### 2.3.1. Particle Size Distribution Test

The particle size of the C-S-H seed agent samples wet grinded for 0 min, 30 min, 60 min, 90 min, and 120 min was measured by Malvern Mastersizer [18], a laser particle size analyzer. Before the measurement, the sample was dissolved in deionized water, ultrasonically dispersed for 5 min, and prepared into a 0.1 g/L solution.

#### 2.3.2. Viscosity Test

According to Chinese national standard GB/T 30514-2014 [35], the viscosity of the C-S-H seed samples doped with 0.5%, 1.0%, 1.5%, 2.0%, 2.5%, and 3.0% PCE was measured.

#### 2.3.3. Fourier Transform Infrared Spectroscopy (FTIR) Test

Nicolet iS20 FTIR was used to analyze the chemical structure of polycarboxylate superplasticizer PCE [36].

#### 2.3.4. Zeta Potential Test

The Zeta potential of the C-S-H seed samples doped with 0.5%, 1.0%, 1.5%, 2.0%, 2.5%, and 3.0% PCE was tested by Malvern potentiostat. Before the measurement, the samples were dissolved in deionized water, ultrasonically dispersed for 5 min, and prepared into a 0.1 g/L solution [37].

#### 2.3.5. X-ray Diffraction (XRD) Test

The hydrates in the C-S-H seed sample powders wet-milled for 0 min, 30 min, 60 min, 90 min, and 120 min and the cement paste sample powders containing 0%, 1%, 3%, 5%, 7%, 9%, and 11% C-S-H seed were analyzed by XRD at a scanning speed of 4°/min. The 2θ range was selected to be 15°–80° [18].

#### 2.3.6. Scanning Electron Microscope (SEM) Test

The microstructure of the samples was observed by SEM. The powders of the C-S-H seed samples with wet grinding times of 0 min, 30 min, 60 min, 90 min, and 120 min and the cement paste sample powders with 0%, 1%, 3%, 5%, 7%, 9%, and 11% C-S-H seed samples were sprayed with Pt, and the voltage during the measurement process was 5 kV [18].

#### 2.3.7. Thermogravimetry–Differential Scanning Calorimetry (TD) Analysis

TD was obtained by a comprehensive thermal analyzer to analyze the C-S-H seed sample powders wet-milled for 0 min, 30 min, 60 min, 90 min, and 120 min and the cement paste sample powders with 0%, 1%, 3%, 5%, 7%, 9%, and 11% C-S-H seed added. The heating rate was 10 °C/min, and the test temperature was 50 °C–800 °C [18].

#### 2.3.8. Concrete Compressive Strength

According to standard GB/T 13476-2023 [33], the press was used to test the 1d compressive strength of concrete with 0%, 1%, 3%, 5%, 7%, 9%, and 11% C-S-H seed. Three samples were measured in each group, and the test average was calculated.

## 3. Results

### 3.1. C-S-H Seed Properties

#### 3.1.1. Particle Size (PS) Distribution

**Figure 2:**
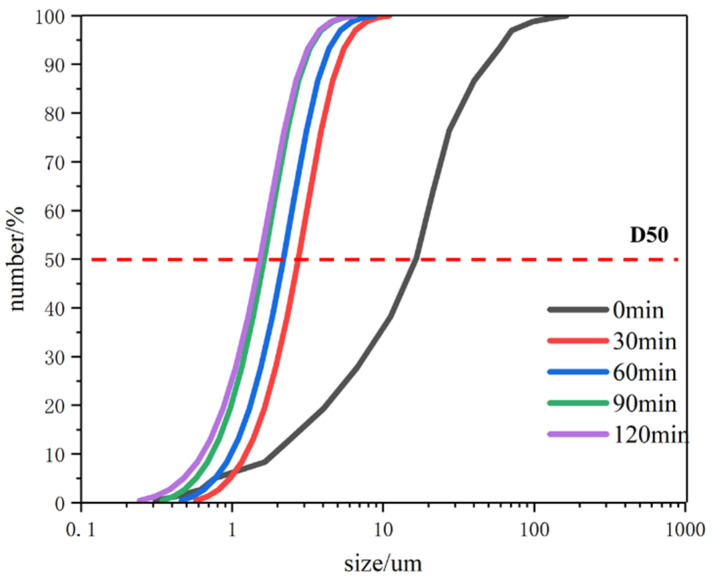
C-S-H seed PS distribution.

The PS distribution of C-S-H seed after grinding for 0 min, 30 min, 60 min, 90 min, and 120 min was measured by a laser PS analyzer, and the results are shown in Figure 2. The PS range of the C-S-H seed after grinding for 0 min was 0.1 μm–200 μm, and the average PS was 16.5 μm; with the increase in grinding time, the PS of the C-S-H seed agent decreased rapidly, and the average PS of the C-S-H seed after grinding for 30 min was 2.7 μm. When the grinding time exceeded 90 min, the PS of the C-S-H seed agent no longer decreased, and the average PS was 1.5 μm. The above results show that the PS of the C-S-H seed agent was greatly refined through wet grinding.

Research has shown that the hydration promoting ability of C-S-H seed is related to PS [38], with smaller particle sizes indicating stronger hydration-promoting ability [9,18]. Under the squeezing, collision, and friction of zirconia balls, the PS of C-S-H seed continuously decreases. However, the PS of the C-S-H seed is affected by the wet grinding process, such as the agitation speed, bead diameter, and bead filling ratio [39,40], which is also the reason why the PS of the C-S-H seed did not decrease after 90 min. The research on wet grinding technology is of great significance for nano-materials and has been widely carried out in the fields of ceramics, medicine, minerals, etc. [41]. However, there are few reports on the study of wet grinding C-S-H seed, and its key parameters are not yet clear. Further research on this is necessary.

**Figure 3:**
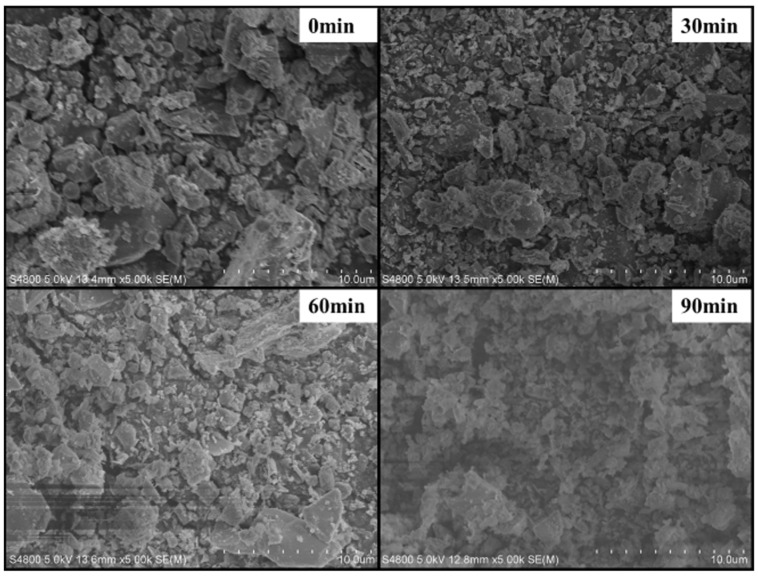
SEM image of C-S-H seed.

The PS of the C-S-H seed after grinding for 0 min, 30 min, 60 min, and 90 min was observed by SEM. It can be seen from Figure 3 that with the increase in grinding time, the PS of C-S-H seed gradually decreased.

#### 3.1.2. FTIR

**Figure 4:**
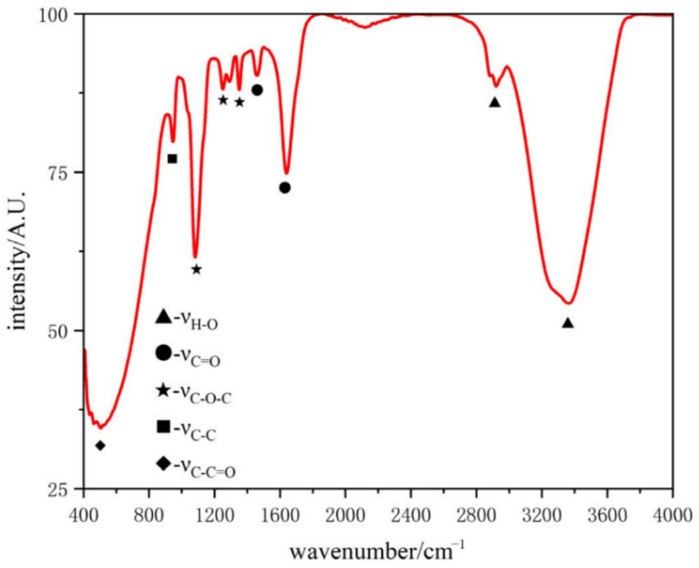
Infrared analysis of PCE.

PCE is the most widely used organic admixture in cement concrete. Polycarboxylate (PCE) has functional groups such as –COOH and –CH_3_O, as shown in Figure 4. Through the σ-electrons and π-electrons of the PCE, the symmetrical vibration peak of the base –OH appeared at 2921 cm^−1^ and 3363 cm^−1^, which was used to identify the –COOH. The C=O symmetrical vibration peak of –COOH appeared near 1640cm^−1^, and the stretching vibration peak of the ether C=O appeared near 1461cm^−1^. In addition, two ester characteristic peaks appeared near 1082cm^−1^ to 1351cm^−1^. In the molecular structure of PCE, the olefinic acid group substituted by acrylic acid constituted the main chain, and polyoxyethylene (PEO) constituted the branch chain to play a role together.

PCE can be adsorbed on the surface of cement particles in large quantities under the action of electrostatic attraction, and these PCE adsorbed on the surface of cement particles can disperse the cement particles through electrostatic repulsion and steric hindrance, thereby improving the dispersion of particles in the slurry and increasing fluidity on a macro scale [42,43,44]. From Figure 4, it can be seen that there was a large amount of –COOH in PCE, which will hydrolyze into –COO–. Studies have shown that the role of PCE in cement is believed to be related to PCE molecules. –COO– in PCE molecules binds to water through hydrogen bonds, forming a layer of water molecules; Ca^2+^ needs to pass through the water molecule layer to bind with –COO– [44]. PCE affects the dispersibility and stability of the C-S-H seed suspensions through its adsorption performance and steric hindrance, which will be discussed in the next section regarding its impact on the viscosity and zeta potential of C-S-H seed.

#### 3.1.3. Viscosity and Zeta Potential

**Figure 5:**
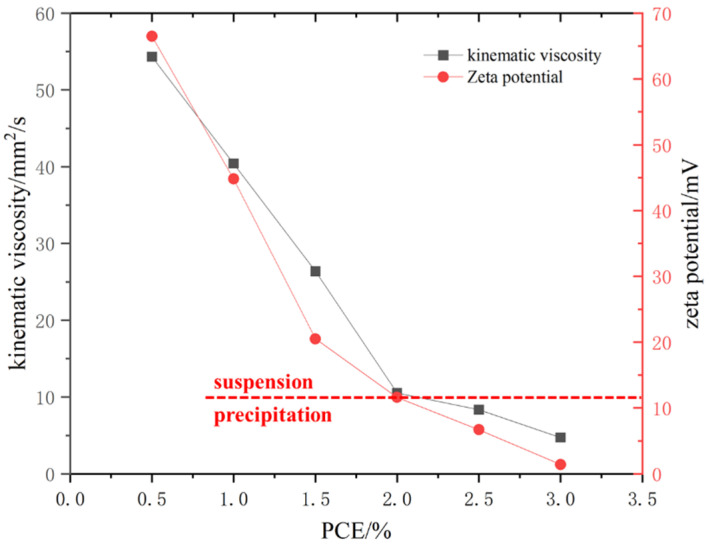
The viscosity and zeta potential of C-S-H seed.

As can be seen from Figure 5, adding PCE can reduce the dynamic viscosity of C-S-H seed and help improve the fluidity of C-S-H seed; with the increase in PCE content, the dynamic viscosity of C-S-H seed first decreased significantly, and then the decline slowed down. The water reducer content in C-S-H seed increased from 0.5% to 1.5% of cement content, and the dynamic viscosity of C-S-H seed decreased significantly, being 21.3%, 48.6%, and 79.5% lower than the proportion of 0.5%. When the PCE content in C-S-H seed exceeded 2%, the dynamic viscosity of C-S-H seed tended to decrease slowly, and the dynamic viscosities of samples with 2.0% and 2.5% PCE content were 17.4% and 16.3% of the sample without PCE, respectively. When the PCE content in the C-S-H seed exceeded 2.5% of the cement content, it was only 9% of the sample without PCE, and the C-S-H seed had good fluidity.

Figure 5 shows the effect of PCE on the zeta potential of C-S-H seed. From the figure below, it can be found that the addition of PCE can cause the absolute value of the zeta potential of C-S-H seed to decrease from the initial 66.5 mV to 1.4 mV. When the amount of PCE in the C-S-H seed was greater than 2%, the zeta potential was less than 20 mV, and the C-S-H seed suspension precipitated. The dispersibility of PCE mainly depends on electrostatic repulsion and steric hindrance. The side chain of PCE can provide stronger steric hindrance, and the presence of –COO– on the main chain causes PCE to have a negative charge.

Although the addition of PCE helps to improve the fluidity of the C-S-H seed agent, the precipitation phenomenon of C-S-H seed becomes more and more obvious with the increase in PCE content. When the PCE content in C-S-H seed did not exceed 2.0%, the C-S-H seed was a stable suspended liquid. With the increase in PCE content, the precipitation amount in C-S-H seed gradually increased, but the precipitation was relatively loose and easy to disperse. When the PCE content in C-S-H seed exceeded 3.0%, the precipitation was not easy to disperse. This is because the adsorption of PCE on the surface of C-S-H seed can change its zeta potential. The decrease in zeta potential can cause the C-S-H seed adsorption liquid to lose stability and precipitation. The results show that the zeta potential of C-S-H seed particles without PCE was positive, while the addition of PCE was able to reduce the zeta potential of cement particles to negative. In addition, after the PCE adsorption reaches the saturated content, the adsorption amount no longer continues to increase, and the corresponding zeta potential of cement particles remains basically unchanged [37,44,45]. Research has shown that the molecular structure of PCE and the –COOH group have a significant impact on the rheological properties and hydration behavior of concrete, making it a hot research direction in PCE [36,46,47]. In this study, PCE affected the viscosity and zeta potential of C-S-H seed suspension by adsorbing C-S-H seeds and the influence of –COOH group on the surface charge of C-S-H seeds. Further research is needed to investigate how the molecular structure of PCE and the –COO– group affect C-S-H seed suspension, which will help design PCE for C-S-H seed suspension.

#### 3.1.4. XRD

**Figure 6:**
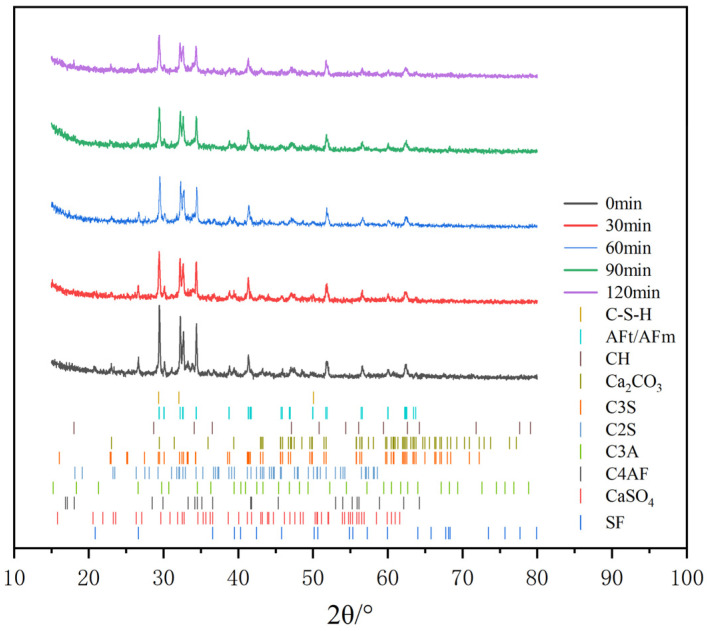
XRD patterns of C-S-H seed at different wet grinding times.

Figure 6 shows the XRD spectra of C-S-H seed ground for 0 min, 30 min, 60 min, 90 min, and 120 min. The C-S-H seed powder ground for 0 min contained C_3_S, C_2_S, C_3_A, C_4_AF, and gypsum of unhydrated cement clinker. The diffraction peak of gypsum appeared at 2θ of 29.5°, 32.6°, and 43.1°; the diffraction peak of C_4_AF appeared at 2θ of 30.2° and 33.8°; and the diffraction peak of C_3_A appeared at 2θ of 29.8° and 61.1°. The diffraction peak of C_2_S appeared at 2θ of 26.5°, 28.4°, and 34.2°, and the diffraction peak of C_3_S appeared at 2θ of 25.5°, 28.1°, and 43.8°. As the wet grinding time increased, the diffraction peaks of C_3_S, C_2_S, C_3_A, C_4_AF, and gypsum decreased, and the diffraction peaks of C-S-H gel, CH, AFm, and CaCO_3_ appeared. These hydration products change the XRD peak curve of the original cement clinker, and they ultimately change the original cement clinker mineral phase and increase with the increase in wet grinding time. The diffraction peaks of C-S-H gel appeared at 2θ of 29.1°, 32.8°, and 50.5°; the diffraction peaks of C-A-H appeared at 2θ of 42.3°; the diffraction peaks of CH appeared at 2θ of 34.6°, 64.7°, and 65.2°; and the diffraction peaks of AFm appeared at 2θ of 43.6°. The C-S-H seed had a large diffuse diffraction peak at about 20°–50°, which is because its microstructure is mainly amorphous.

The phase characteristics of C-S-H seed analyzed by XRD after wet grinding 0 min, 30 min, 60 min, 90 min, and 120 min are displayed in Figure 6. The results show that the diffraction peaks of C3S, C2S, C3A, C4AF, and gypsum and other substances in the XRD pattern of the 30 min, 60 min, 90 min, and 120 min C-S-H seed were significantly lower than those of the 0 min one. This depicted that the crystalline phase in C-S-H seed after wet grinding was significantly reduced. The reason was that wet grinding forms a new type of amorphous phase or was transformed into an amorphous phase due to crystallization, which means that the hydration reaction of cement produces C-S-H gel [18].

#### 3.1.5. TD

**Figure 7:**
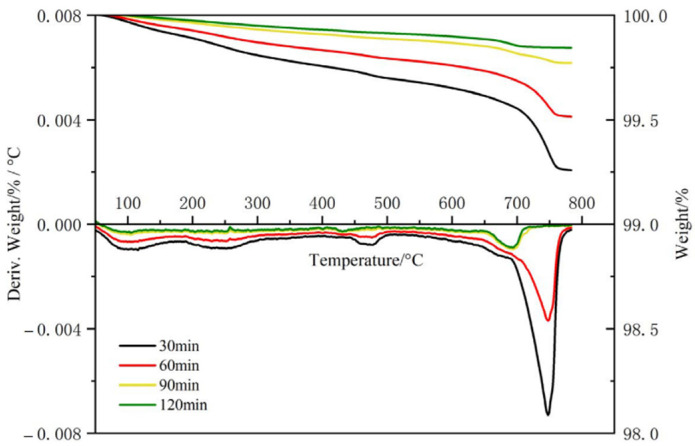
TD spectra of C-S-H seed at different wet grinding times.

TD is widely used in the detection of cement-based materials. It mainly measures the mass loss of cement hydration samples due to chemical changes such as dehydration and thermal decomposition of products within a fixed temperature range. This method mainly determines the content of hydration products in a semi-quantitative form, which effectively reflects the difference in the degree of hydration of different samples. The effect of wet grinding time on the hydration degree of silicon C-S-H seed was studied by TD. The results are shown in Figure 7.

In the TD curve of the sample, there were multiple exothermic peaks, one of which was between 50 °C and 100 °C, which is mainly the dehydration peak of calcium sulfonate and a small amount of unremoved free water. Another peak was between 100 °C and 200 °C, which is mainly the dehydration peak of C-S-H gel, calcium sulfonate, and residual gypsum. At about 460 °C, it was the decomposition peak of CH; at 550 °C–800 °C, it was the endothermic peak generated by the decomposition of the hydration products of CaCO_3_.

As can be seen from Figure 7, with the increase in grinding time, the hydration products in the C-S-H seed agent gradually increased. The results show that the mass loss of C-S-H seeds after 90 min of wet grinding was reduced by 80% compared to the 30 min one at 400 °C–500 °C. As the wet grinding time increased, the content of CH in C-S-H seeds decreased significantly. This is because during the wet grinding process of cement, gypsum and C_3_A dissolve first, forming AFt/AFm as well as Ca^2+^ and OH^−^. With the further hydration of cement, Ca^2+^ and SiO_4_^3−^ combine to form C-S-H gel, and Ca^2+^ is consumed [9,10]; therefore, the DT results show that the content of CH in C-S-H gel decreased with the increase in wet grinding time. And this result is consistent with the XRD result. Generally, cement takes weeks or even longer to be fully hydrated [14], and the wet grinding process can promote cement hydration, which can be completed within a few hours.

### 3.2. Concrete Properties

#### 3.2.1. Compressive Strength

**Figure 8:**
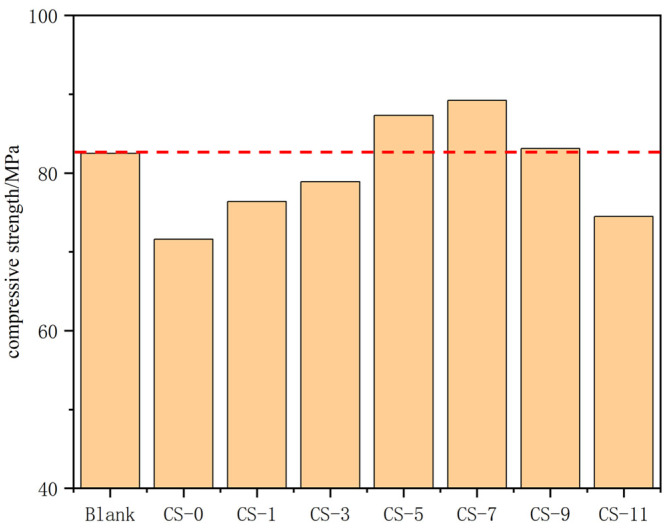
Compressive strength.

The results of the compressive strength of concrete with different C-S-H seed contents are shown in Figure 8. The strength of concrete increased first and then decreased with the increase in the C-S-H seed content. The concrete strength was the highest when the C-S-H seed content was 5%~7%. Adding C-S-H seeds to high-strength concrete can replace part of the cement with silica fume without affecting the strength of the concrete, thereby reducing the amount of cement. Compared with the benchmark, when silica fume replaced 21.8% cement, the strength of the concrete decreased by 21.2%. This is because the hydration activity of silica fume is poor, and it needs to react with CH produced by cement hydration to generate C-S-H gel to provide strength for the concrete. Therefore, a large amount of silica fume is used in concrete, which requires a longer curing time [48,49]. When the C-S-H seed content was greater than 5%, the concrete strength increased by 10%. This is because C-S-H seeds can provide C-S-H seed sites in cement paste, reduce C-S-H seed potential energy, and reduce the thickness of the hydration layer, thereby accelerating cement hydration [14]. And a large amount of CH is produced through cement hydration, which reacts with silica fume to form C-S-H gel, making the concrete more compact and blocking the interconnected pores in the concrete, thereby improving the concrete strength. When the C-S-H seed content was greater than 7%, the concrete strength decreased. This is because too much C-S-H seeds increase the unit water demand of concrete. Research has shown that nano early strength agents can promote cement hydration; however, they have adverse effects on the fluidity and workability of concrete, because the larger specific surface area of nanomaterials would increases the internal friction force of concrete and also adsorb more water, resulting in a lack of water lubrication that increases friction between solid particles in concrete [50,51,52,53]. When the content of C-S-H seeds in concrete exceeded 7%, due to the high fineness of C-S-H seeds, a large amount of water was adsorbed, resulting in a decrease in the workability of the concrete. When the water–cement ratio remained unchanged, the greater the C-S-H seed content, the worse the concrete workability, which affected the structure of the concrete, formed harmful pores in the concrete, and reduced the concrete strength.

#### 3.2.2. XRD

**Figure 9:**
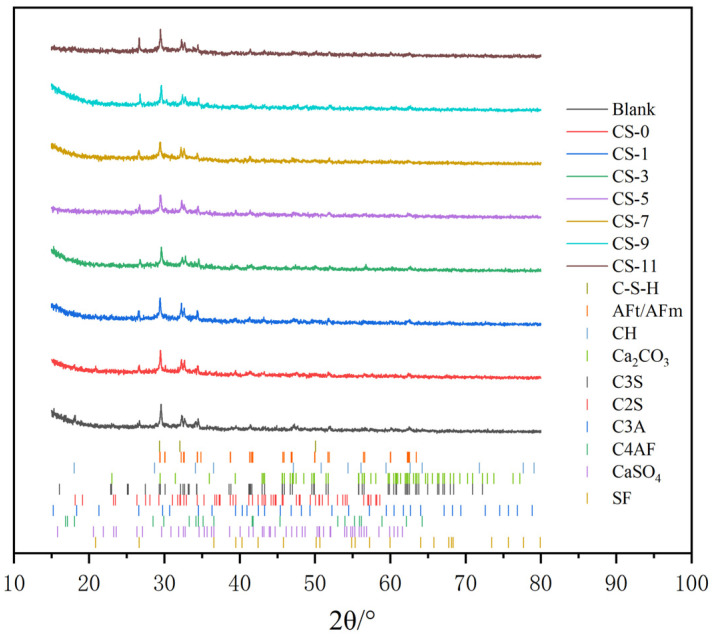
XRD of concrete with different C-S-H seed contents.

Figure 9 shows the XRD spectra of the base and concrete with 0%, 1%, 3%, 5%, 7%, 9%, and 11% CSH seed added. The diffraction peaks of C-S-H gel, CH, AFm, and CaCO_3_, as well as C_3_S, C_2_S, C_3_A, C_4_AF, and gypsum of a small amount of unhydrated cement clinker are shown. Compared with the blank, CS-0 had lower diffraction peaks of C-S-H gel at 32.8° and AFm at 43.2°, and it had higher diffraction peaks of C_3_S at 30.5°, C_2_S at 32.5°, C_3_A at 38.2°, and C_4_AF at 26.4°. This indicates that its hydration degree was lower than that of the blank. The lower CH peaks at 27.6° indicate that its reaction consumed the CH produced by cement hydration, but the CH produced by cement hydration was not enough to excite all silica fume. After adding C-S-H seed, the peak intensity of the diffraction peaks of C-S-H gel, AFm, and CaCO_3_ in concrete increased with the increase in C-S-H seed content, which indicates that adding C-S-H seed to concrete can accelerate cement hydration. The peak intensity of C_3_S, C_2_S, C_3_A, C_4_AF, and gypsum was weakened, which also indicates that C-S-H seed promotes the formation of hydration products. Combined with the mechanical properties test, it can be seen that when the C-S-H seed agent dosage exceeded 7%, although the C-S-H seed promoted the hydration of cement in concrete, the peak intensities of the diffraction peaks of C-S-H gel, AFm, and CaCO_3_ were significantly enhanced. However, too much C-S-H seed increases the unit water demand of concrete, which leads to worse workability of concrete, lower internal density of concrete, more harmful pores, and lower strength.

**Figure 10:**
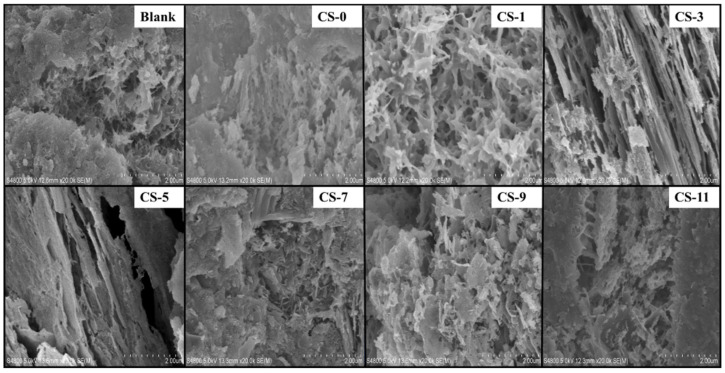
SEM of concrete with different C-S-H seed contents.

It can be seen from Figure 10 that with the increase in C-S-H seed content, the C-S-H gel in the concrete increased and was cross-linked to form a network. When the C-S-H seed content exceeded 5%, the C-S-H gel in the concrete further increased to form a high-density continuous solid.

The strength of concrete mainly comes from the crosslinking and stacking of C-S-H gel. Studies have shown that cement can provide growth sites through cement particles or C-S-H seeds, and hydration generates C-S-H gel. Different growth methods have a significant impact on the morphology of C-S-H gel [14,53]. From Figure 9 and Figure 10, it can be seen that adding C-S-H seeds can promote cement hydration. With the increase in C-S-H seed content, a large number of C-S-H gels are generated through C-S-H seeds in the gaps between cement particles, which helps to crosslink and stack between C-S-H gels, forming high-density C-S-H from low-density C-S-H [54], thereby improving concrete strength.

#### 3.2.3. TD

**Figure 11:**
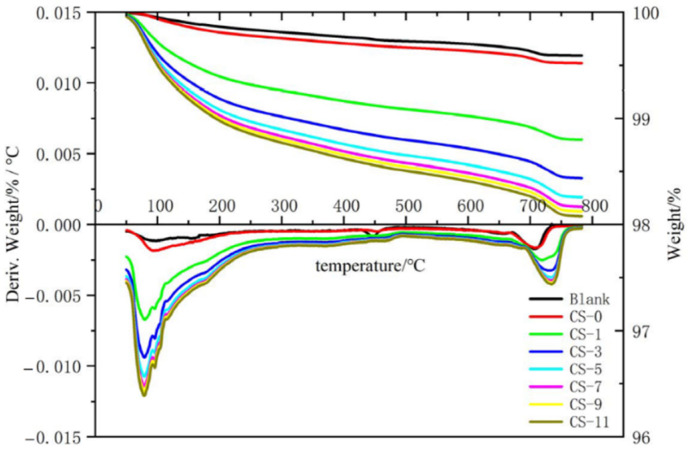
TD spectrum of concrete with different C-S-H seed contents.

The effect of wet grinding time on the hydration degree of silicon seed agent was studied by TD, and the results are shown in Figure 11. As shown in the figure, there were multiple exothermic peaks in each TG curve. One peak was between 50 °C and 100 °C, which was mainly the dehydration peak of calcium sulfonate and a small amount of unremoved free water. Another peak was between 100 °C and 200 °C, which was mainly the dehydration peak of C-S-H gel, calcium sulfonate, and residual gypsum. At about 460 °C, it was the decomposition peak of CH; at 550 °C–800 °C, it was the endothermic peak generated by the decomposition of the hydration product of CaCO_3_. As can be seen from the figure, at 50 °C–200 °C, the quality loss of concrete increased with the increase in C-S-H seed content, because the addition of C-S-H seed helps cement hydration and produces more C-S-H gel and AFt/AFm [11,14]; the more C-S-H seed is added, the more hydration products are produced. Meanwhile the blank group lost less weight than others at 400 °C–500 °C, that is, because SF depleted CH and gained C-S-H gel. With the increase in the amount of C-S-H seed, the weight loss increased significantly, indicating that the hydration products gradually increased, which is consistent with the XRD results. It is confirmed that when the C-S-H seed content exceeded 7%, although the C-S-H seed promotes the hydration of cement in concrete, the peak intensities of the diffraction peaks of C-S-H gel, AFm, and CaCO_3_ were significantly enhanced. However, the corresponding compressive strength did not increase, but instead decreased, because nano early strength agents have adverse effects on the fluidity and workability of concrete [47,48,49], and too much C-S-H seed increases the unit water demand of concrete, which leads to worse workability of concrete, lower internal density of concrete, more harmful pores, and lower strength.

## 4. Conclusions

The conclusions are as follows:

1. The wet grinding process can reduce the PS of C-S-H seed and promote its hydration degree. With the increase in wet grinding time, the PS of the C-S-H seed agent continued to decrease, and the hydration degree continued to increase. When the wet grinding time exceeded 90 min, the PS and hydration degree of the C-S-H seed agent no longer increased. There are few reports on the study of wet grinding of C-S-H seed, and its key parameters are not yet clear. Further research on this is necessary.
2. The addition of PCE can reduce the dynamic viscosity of the C-S-H seed suspension and make it have good fluidity. When the amount of PCE increased to 1.5%, the dynamic viscosity of the C-S-H seed suspension was significantly reduced. When the amount exceeds 2%, the dynamic viscosity decreased slowly, that is, the amount of PCE in the C-S-H seed suspension should not be less than 1.5%.
3. PCE can cause the C-S-H seed suspension to agglomerate and precipitate; With the increase in the amount of PCE, the zeta potential of the C-S-H seed gradually decreased. When the amount of PCE in the crystal seed was greater than 2%, the zeta potential was less than 20 mV, precipitate formed in the C-S-H seed suspension, and the amount of PCE in the C-S-H seed suspension should not be greater than 2.0%. PCE affects the viscosity and zeta potential of C-S-H seed suspension by adsorbing C-S-H seeds and the influence of –COOH group on the surface charge of C-S-H seeds. Further research is needed to investigate how the molecular structure of PCE and the –COO– group affect C-S-H seed suspension, which will help design PCE for C-S-H seed suspension.
4. C-S-H seed can improve the early performance of concrete while adding silica fume to replace cement, making the concrete denser and improving the strength of concrete. When the amount of C-S-H seed agent was less than 5%, the concrete strength increased less. When the amount of C-S-H seed agent was higher than 7%, due to the small particle size of C-S-H seed, this will increase the unit water consumption of concrete, making the concrete workability worse and causing the concrete strength to decrease. Therefore, the amount of C-S-H seed agent should be controlled at 5%~7%.

## Figures and Tables

**Table 1:** XRF of raw materials (%).

Sample	SiO_2_	Al_2_O_3_	CaO	Fe_2_O_3_	SO_3_	MgO	LOI
Cement	22.84	4.53	61.98	3.41	2.83	2.61	1.80
GGBS	34.13	13.52	40.88	0.51	2.36	6.79	1.81
SF	98.31	0.14	0.56	0.21	0.12	0.31	0.35

**Table 2:** Concrete mix ratio (kg/m^3^).

Sample	Cement	GGBS	SF	Sand	Aggregate	Water	C-S-H Seed
Blank	320	85	40	620	1380	110	0
CS-0	250	85	110	620	1380	110	0
CS-1	246	85	110	620	1380	110	18
CS-3	237	85	110	620	1380	110	54
CS-5	228	85	110	620	1380	110	89
CS-7	219	85	110	620	1380	110	125
CS-9	210	85	110	620	1380	110	160
CS-11	201	85	110	620	1380	110	196

Note: C-S-H seed suspension contains 75% moisture; deduct the water content of C-S-H seed agent from the water used in concrete to keep the total water content of concrete unchanged.

**Table 3:** Cement paste mix ratio (kg/m^3^).

Sample	Cement	GGBS	SF	Water	C-S-H Seed
Blank	320	85	40	110	0
CS-0	250	85	110	110	0
CS-1	246	85	110	110	18
CS-3	237	85	110	110	54
CS-5	228	85	110	110	89
CS-7	219	85	110	110	125
CS-9	210	85	110	110	160
CS-11	201	85	110	110	196

Note: C-S-H seed suspension contains 75% moisture; deduct the water content of the C-S-H seed from the water used in the cement paste to keep the total water content of the cement paste unchanged.

## Data Availability

All data are contained within the article.
